# Advances in the Biology, Ecology, and Management of Plant Pests

**DOI:** 10.3390/biology15120901

**Published:** 2026-06-09

**Authors:** Yu Gao, Suli Liu, Yuxin Zhou, Lei Bian

**Affiliations:** 1 College of Plant Protection, Jilin Agricultural University, Changchun 130118, China; gaothrips@jlau.edu.cn (Y.G.);; 2 School of Mathematics, Jilin University, Changchun 130012, China; 3 Tea Research Institute, Chinese Academy of Agricultural Sciences, Hangzhou 310008, China

## 1. Introduction

Plant pests pose a severe threat to global agricultural productivity and ecosystem stability [1,2]. Each year, crop losses caused by pests amount to hundreds of billions of dollars, gravely endangering food security and farmers’ livelihoods [3]. Meanwhile, although traditional chemical control methods are effective in the short term, they face increasingly serious challenges: The rapid evolution of pest resistance leads to escalating pesticide use with diminishing returns and chemical residues contaminate soil and water sources, damaging the ecological environment [4]; moreover, broad-spectrum insecticides inflict fatal harm on beneficial non-target organisms such as bees and ladybugs, disrupting ecological balance [5]. Confronted with these dilemmas, the development of green and sustainable pest management strategies has become an urgent global agricultural necessity [6]. Environmentally friendly technologies, such as biological control [7] and pest-resistant crop breeding [8], are emerging as critical pathways to replace traditional chemical control and achieve sustainable agriculture.

The nine contributions (eight articles and one editorial) in this Special Issue, ‘Biology, Ecology, and Management of Plant Pests’, showcase cutting-edge advances in plant pest research across three integrated dimensions: biology, ecology, and the management of plant pests. Biology encompasses the molecular basis of chemosensory systems and the physiological toxicity and molecular response mechanisms of plant essential oils. Ecology addresses multi-trophic interactions and environmental adaptation, including the thermal plasticity of parasitoid wasps, plant–pollinator volatile signaling, microbiome dynamics of scale insects, and microbe-mediated kairomone systems between noctuid hosts and their parasitoids. Management focuses on practical tools and strategies, spanning species-specific molecular diagnostics for thrips, and the environmental risk assessment of RNAi-based transgenic cotton for non-target predators.

## 2. Biology of Plant Pests

The following two articles, from the perspectives of the molecular basis of chemosensory systems and the physiological toxicity and molecular response mechanisms of plant essential oils, respectively, reflect the fundamental research on physiological mechanisms, molecular foundations, and other aspects of pests within the framework of the biology of plant pests.

Shi et al. conducted transcriptome analysis across multiple adult tissues of the soybean pod borer *Leguminivora glycinivorella* to identify chemosensory genes [9]. A comprehensive repertoire was annotated, including 76 odorant receptors (ORs), 15 gustatory receptors (GRs), 18 ionotropic receptors (IRs), 52 odorant-binding proteins (OBPs), 18 chemosensory proteins, and four sensory neuron membrane proteins (SNMPs). Notably, canonical bitter GRs and specific IR lineages (IR100/IR85a) were absent, potentially reflecting the adaptation to specialized soybean-feeding. Pronounced sexual and tissue dimorphism was observed: male antennae were enriched for putative pheromone receptors and *LglySNMP1*, while female-biased OBPs and ORs suggested roles in host location and oviposition. This study provides a valuable expression atlas for developing behavior-based green pest management strategies.

Wang et al. (2025) evaluated the insecticidal activity of *Angelica archangelica* root essential oil (AAEO) against the maize weevil *Sitophilus zeamais* through gas chromatography–mass spectrometry (GC-MS) analysis, fumigation toxicity assays, enzyme activity measurements, and transcriptome sequencing [10]. GC-MS identified 35 components, with δ-3-Carene, Limonene, and α-Pinene as major constituents. The LC_50_ values were 164.38, 132.62, and 90.35 mg/L air at 24, 48, and 72 h, respectively. AAEO significantly inhibited acetylcholinesterase, glutathione S-transferase, and carboxylesterase activities. RNA-seq revealed 3718 significantly differentially expressed genes, with qRT-PCR confirming the reliability of the transcriptome data. This study provides a scientific basis for developing effective plant essential oil-based bioinsecticides against stored-grain pests.

## 3. Ecology of Plant Pests

The following four articles from the perspectives of temperature-driven natural enemy population dynamics and climate adaptation, multi-trophic chemical information transfer and microbe-mediated interaction networks, plant–pollinator chemical communication and community function optimization, and pest microbiome community structure and host–plant interactions, respectively, reflect the core essence of ecological-level research in the ecology of plant pests, encompassing pest–environment interactions, population dynamics, community structure, food-web relationships, and climate adaptation, while also laying a theoretical foundation for the development of green pest control technologies.

The study by Wang et al. (2025) systematically evaluated the effects of temperature (21–33 °C) on the parasitism capacity and reproductive fitness of *Sclerodermus guani* [11]. Results showed that temperature significantly affected female wasp parasitoid behavior, the pre-oviposition period, and offspring developmental duration: as the temperature increased, host-seeking and stinging behaviors became more active, and the pre-oviposition period shortened from 31.3 days (21 °C) to 4.1 days (33 °C). Reproductive fitness peaked at 27–30 °C, with both low and high temperatures being detrimental; offspring numbers decreased by 30% at 33 °C compared to the maximum. The developmental threshold temperatures for the egg, larval, and pupal stages were calculated as 10.19 °C, 7.73 °C, and 15.57 °C, respectively. This study provided biological data above 30 °C for the first time, offering important guidance for breeding heat-tolerant strains and optimizing indoor rearing and field release strategies under climate change.

The study by Wang et al. (2025) investigated the relative attractiveness of the host larval body and frass volatiles to the parasitoid *Microplitis mediator*, using two noctuid pests (*Helicoverpa armigera* and *Spodoptera frugiperda*) as hosts [12]. Through four-arm olfactometer bioassays, GC-MS analysis, and gas chromatography–electroantennographic detection (GC–EAD), larval frass extracts were found to be significantly more attractive to parasitoids than body extracts, and this attractiveness was independent of host food types (maize, cotton, soybean leaves, or artificial diet). Ethyl palmitate was identified as the key attractant compound in frass, eliciting strong antennal responses and behavioral attraction in parasitoids. Antibiotic treatment experiments indicated that ethyl palmitate production was associated with gut bacterial metabolism. This study revealed for the first time that a ubiquitous gut microbe-derived metabolite in noctuid larval frass serves as a critical infochemical for parasitoid host location, providing novel insights for enhancing biological control using volatile attractants.

The study by da Silva et al. investigated changes in the microbiota of the armored scale insect *Diaspis echinocacti* infesting *Opuntia stricta cladodes* across low, intermediate, and high infestation levels using 16S rRNA amplicon sequencing [13]. The obligate endosymbiont *Candidatus Uzinura* diaspidicola dominated all samples, indicating a resilient core microbiome. Shannon diversity showed a declining trend from low to high infestation, and PICRUSt2 predicted a contraction of metabolic breadth at higher infestations with convergence on energy and amino acid biosynthesis pathways. This study provides baseline data on scale insect microbiota dynamics and their potential role in host–plant interactions, guiding future microbiome-based strategies for sustainable cactus protection.

The study of He et al. (2025) identified 32 volatile compounds from Changbai Mountain blueberry flowers using solid-phase microextraction (SPME) combined with GC-MS, with linalool and styrene as the primary components [14]. Through electroantennography and Y-tube olfactometer behavioral assays, six compounds with significant electrophysiological activity in bumblebees were screened (benzaldehyde, phenylpropylaldehyde, citral, linalool, α-terpineol, and geraniol). The study found that low concentrations of geraniol, linalool, and α-terpineol attracted bumblebees, while high concentrations of benzaldehyde, phenylpropylaldehyde, and citral showed repellent effects. This research systematically elucidated the chemical interaction mechanism between blueberry floral volatiles and bumblebees for the first time, providing a theoretical basis for developing natural pollination attractants to improve blueberry yield and quality.

## 4. Management of Plant Pests

The following two articles from the perspectives of molecular monitoring technology development and application and the ecological risk assessment and safety management of genetically modified crops, respectively, reflect the core essence of applied research in Integrated Pest Management, encompassing pest monitoring, control technology development, risk assessment, and comprehensive management strategies. They provide important technical support and theoretical foundations for accurate pest identification, early warning, and sustainable management.

Qiao et al. developed a species-specific *CO*I PCR approach for discriminating four co-occurring thrips species (*Frankliniella intonsa*, *F. occidentalis*, *Megalurothrips usitatus*, and *Thrips hawaiiensis*) using crude DNA extracts [15]. By designing four species-specific primer pairs targeting polymorphism-rich regions of the mitochondrial *CO*I gene with diagnostic 3′-terminal nucleotides, combined with a PBS-based short-turnaround DNA extraction workflow, the assay achieved a practical detection limit of 1 ng per reaction and maintained reproducible amplification under mixed-species backgrounds. PCR inhibition from crude lysates was effectively alleviated by fivefold dilution. This framework provides a field-adaptable tool for thrips species identification and invasive species monitoring, with potential for integration with visual nucleic acid detection platforms.

The study of Yao et al. (2025) systematically assessed the potential ecological risks of dsAsFAR transgenic cotton targeting *Adelphocoris suturalis* on the non-target predatory lady beetle *Harmonia axyridis* [16]. Through two exposure pathways—direct artificial diet feeding and trophic transfer—combined with transcriptome sequencing and qRT-PCR validation, results showed that dsAsFAR had no significant adverse effects on lady beetle growth, development, survival, predation ability, fecundity, or FAR homologous gene expression. Although a few off-target differentially expressed genes were detected, Shannon entropy analysis indicated that the overall transcriptome homeostasis was not disturbed. This study established a comprehensive risk assessment framework including exposure pathway evaluation, worst-case feeding trials, off-target effect analysis, and transcriptome stability assessment, providing an important reference for the biosafety evaluation of RNAi transgenic crops.

This Special Issue covers the biology, ecology, and management of plant pests, spanning chemosensory mechanisms, essential oil insecticidal activity, parasitoid thermal plasticity, microbe-mediated kairomones, scale insect microbiome dynamics, blueberry-pollinator signaling, thrips molecular diagnostics, and RNAi cotton risk assessment, offering key foundations for green pest management. Future work should harness multi-omics and AI to map pest–plant–enemy–microbe networks and pinpoint new control targets [17,18,19]. Sharper predictive models for pest adaptation and resistance under climate change, plus the field-scale deployment of RNAi biopesticides, pheromone traps, and microbial agents, will prove vital [20,21]. Building an integrated management system rooted in ecological regulation and smart monitoring can thus secure both agricultural sustainability and ecological safety.

## Data Availability

The original contributions presented in this study are included in the list of contributions. Further inquiries can be directed to the corresponding authors.
